# Surgical masks and filtering facepiece class 2 respirators (FFP2) have no major physiological effects at rest and during moderate exercise at 3000-m altitude: a randomised controlled trial

**DOI:** 10.1093/jtm/taad031

**Published:** 2023-03-06

**Authors:** Giovanni Vinetti, Alessandro Micarelli, Marika Falla, Anna Randi, Tomas Dal Cappello, Hannes Gatterer, Hermann Brugger, Giacomo Strapazzon, Simon Rauch

**Affiliations:** Institute of Mountain Emergency Medicine, Eurac Research, Bolzano, Italy; Institute of Mountain Emergency Medicine, Eurac Research, Bolzano, Italy; Institute of Mountain Emergency Medicine, Eurac Research, Bolzano, Italy; Center for Mind/Brain Sciences (CIMeC), University of Trento, Rovereto (TN), Italy; Department of Neurology, General Hospital of Bolzano, Bolzano, Italy; Institute of Mountain Emergency Medicine, Eurac Research, Bolzano, Italy; Center for Mind/Brain Sciences (CIMeC), University of Trento, Rovereto (TN), Italy; Institute of Mountain Emergency Medicine, Eurac Research, Bolzano, Italy; Institute of Mountain Emergency Medicine, Eurac Research, Bolzano, Italy; Institute for Sports Medicine, Alpine Medicine and Health Tourism (ISAG), UMIT TIROL-Private University for Health Sciences and Health Technology, Hall in Tirol, Austria; Institute of Mountain Emergency Medicine, Eurac Research, Bolzano, Italy; Institute of Mountain Emergency Medicine, Eurac Research, Bolzano, Italy; Institute of Mountain Emergency Medicine, Eurac Research, Bolzano, Italy; Department of Anaesthesiology and Intensive Care Medicine, Hospital of Merano (SABES-ASDAA), Merano (BZ), Italy; Lehrkrankenhaus der Paracelsus Medizinischen Privatuniversität

**Keywords:** Personal protective equipment, high altitude, hypobaric chamber, arterial blood gas, cognition, COVID-19

## Abstract

**Background:**

During the COVID-19 pandemic, the use of face masks has been recommended or enforced in several situations; however, their effects on physiological parameters and cognitive performance at high altitude are unknown.

**Methods:**

Eight healthy participants (four females) rested and exercised (cycling, 1 W/kg) while wearing no mask, a surgical mask or a filtering facepiece class 2 respirator (FFP2), both in normoxia and hypobaric hypoxia corresponding to an altitude of 3000 m. Arterialised oxygen saturation (SaO_2_), partial pressure of oxygen (PaO_2_) and carbon dioxide (PaCO_2_), heart and respiratory rate, pulse oximetry (SpO_2_), cerebral oxygenation, visual analogue scales for dyspnoea and mask’s discomfort were systematically investigated. Resting cognitive performance and exercising tympanic temperature were also assessed.

**Results:**

Mask use had a significant effect on PaCO_2_ (overall +1.2 ± 1.7 mmHg). There was no effect of mask use on all other investigated parameters except for dyspnoea and discomfort, which were highest with FFP2. Both masks were associated with a similar non-significant decrease in SaO_2_ during exercise in normoxia (−0.5 ± 0.4%) and, especially, in hypobaric hypoxia (−1.8 ± 1.5%), with similar trends for PaO_2_ and SpO_2_.

**Conclusions:**

Although mask use was associated with higher rates of dyspnoea, it had no clinically relevant impact on gas exchange at 3000 m at rest and during moderate exercise, and no detectable effect on resting cognitive performance. Wearing a surgical mask or an FFP2 can be considered safe for healthy people living, working or spending their leisure time in mountains, high-altitude cities or other hypobaric environments (e.g. aircrafts) up to an altitude of 3000 m.

## Introduction

During the Coronavirus disease 2019 (COVID-19) pandemic, the World Health Organisation as well as various national governments advised citizens to wear face masks in public and at work.^1^ The use of filtering facepiece class 2 respirators (FFP2) has been recommended to health workers^2^ including mountain rescuers,^3^ as well as to other professional groups and in high-risk settings depending on local regulations.

Wearing face masks mitigates the transmission of SARS-CoV-2 and other airborne pathogens^4^ but can also have side effects. At sea level, they were mainly related to mild hypercapnia and, particularly during exercise, slight oxygen desaturation.^5–7^ The effect on arterial oxygen partial pressure (PaO_2_) and oxygen saturation (SaO_2_) may be of particular importance at high-altitude or in other hypobaric environments (e.g. aircrafts), since at lower PaO_2_, due to the sigmoid shape of the oxyhaemoglobin dissociation curve, small changes in PaO_2_ lead to more significant changes in SaO_2_.^8^ These parameters were not affected by face masks in resting conditions during a simulated commercial flight at a cabin pressure corresponding to 2286 m above sea level,^9^ a reassuring finding given their proven efficacy in protecting from in-flight infections.^10^ However, it is not known whether mask wearing at higher altitudes, either at rest or during physical activity, may influence blood gases to a greater extent. In such circumstances, hypoxaemia may impair physical^11^ and cognitive performance,^12–14^ negatively affecting work performance and occupational health, as well as increasing the risk of accidents. Besides hypoxaemia, wearing a mask may cause discomfort, affect wellbeing and challenge heat dissipation during exercise or hot weather conditions (while hypoxia further increases perceived exertion), which in turn may interfere with work performance or wearing adherence.^15^ All those parameters are of particular relevance for workers (e.g. health practitioners), travellers and residents in high altitude areas as well as for aircraft passengers or crew.

The present study aims to investigate whether surgical masks and FFP2 worn during a short time period in hypobaria equivalent to an altitude of ~3000 m affect gas exchange, tissue oxygenation, perceived discomfort and dyspnoea at rest and during moderate exercise, as well as cognitive performance at rest and body temperature during exercise. We hypothesised that at high-altitude mask wearing may have a significant effect on those physiological parameters, especially in combination with exercise.

## Methods

### Participants

Eight participants (four females) aged 30 ± 6 years (range 23–40), resident at low altitude (median 231 m, range 21–1100) gave written informed consent to participate and completed the study. Sample size corresponded to the number of matched pairs required to detect a 2 mmHg and 2% mean difference in PaCO_2_ and SaO_2_, respectively, assuming for the standard deviations the same values as the mean differences, a Type I error of 0.05 and a statistical power of 0.8. Participants had sedentary occupations and participated in heterogeneous leisure activities but were neither regular mountaineers nor exposed to high altitude in the preceding month. All were non-smokers, non-obese (174 ± 8 cm, 71 ± 10 kg, BMI 23 ± 3 kg m^−2^, range 19–27), with no history of systemic disease, normal physical examination, electrocardiogram, oxygen saturation, spirometry and ear-nose-throat examination (including anterior rhinoscopy, oro-pharyngoscopy, fibre-optic laryngoscopy). The study was conducted in accordance with the Declaration of Helsinki and was approved by the Ethics Committee for Clinical Trials and Testing of the South Tyrol Health Authority (No. 65–2021).

### Study design

**Figure 1:**
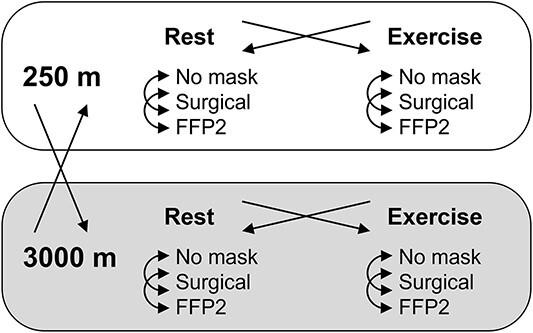
Study design. Arrows indicate randomisation and crossover

The study design was a randomised crossover trial (Figure 1). Participants rested and exercised while wearing either no mask, a surgical mask or an FFP2, both in normoxia (NX) and in hypobaric hypoxia (HH). Rest-exercise, mask and altitude sequence was randomised and balanced between participants, but in each subject rest-exercise and mask sequence was kept the same in NX and HH to better evaluate the effect of altitude. Participants were blinded to the altitude. Participants had a light meal before both the NX and HH sessions, which were separated by a 2-h washout and completed the protocol in one day.

### Interventions

The whole protocol was conducted inside an environmental chamber (terraXcube, Eurac Research, Bolzano, Italy) and was replicated in both NX (250 m altitude, barometric pressure 744 ± 3 mmHg) and HH (ambient pressure 526 ± 0 mmHg), corresponding to an altitude of 3000 m according to the International Civil Aviation Organisation (ICAO) Standard Atmosphere, and 3170 m according to the Model Atmosphere.^16^ Decompression and recompression rate was 3 m s^−1^. To ensure blinding, in NX ascent and descent were simulated by means of decompression-recompression cycles of similar duration. Temperature and humidity were kept stable at 22.0 ± 0.2 °C and 45 ± 3%. After the ascent, subject rested for an additional 20 min without mask before starting the first session. Investigated face masks were (i) a type IIR surgical mask, Class I medical device, EN 14683:2019 (Functional Srl, Bolzano, Italy) and (ii) a 3-layer FFP2, EN 149:2001 + A1:20093 (Functional Srl, Bolzano, Italy). Participants repeated each exercise and rest session three times (one for each mask condition, in random order), with a 15-min washout without mask in between. During rest sessions, each subject completed a cognitive test battery lasting ~10 min as detailed afterwards. Exercise sessions consisted of 12-min cycling at 60 revolutions min^−1^ on a cycle ergometer (E100, COSMED, Rome, Italy), whose saddle height was individually adjusted and recorded. Power output was set at a 1 W kg^−1^, approximately that of hiking at 3000 m,^17^ thus within the moderate intensity domain, which is also the most prevalent intensity of occupational physical activities.^18^ Five minutes of habituation to the assigned mask were allowed both at rest, before starting the cognitive test, and at the beginning of exercise, during which measurements were discarded and mask fitting could be adjusted by the subject or the operator.

### Physiological measurements

Arterialised earlobe capillary blood samples were drawn between minute 10:00 and 12:00 of exercise, and immediately after the end of the cognitive test battery. Carbon dioxide partial pressure (PaCO_2_), PaO_2_, SaO_2_, pH, bicarbonate ([HCO_3_^−^]) and lactate concentration ([La]) were measured with a blood gas analyser (ABL 90 Flex, Radiometer, Brønshøj, Denmark). Heart rate (HR), respiratory rate (*f*_R_) and forehead peripheral oxygen saturation (SpO_2_) (8000R, Nonin Medical, Plymouth, MN, USA) were acquired by an integrated wearable monitoring system (EQ02, Equivital, Cambridge, UK). Left cerebral frontal lobe oxygenation (ScO_2_) was measured with a near infrared spectroscopy device (O3, Masimo Corporation, Irvine, CA, USA). At rest, those continuous measurements were averaged during the whole duration of the cognitive test battery, while during exercise they were averaged from minute 5:00 to 10:00 (to avoid possible perturbations of the subsequent blood sampling). Bilateral tympanic temperature (T_Ty_) was monitored with thermistors placed in the ear canals close to the eardrum (TTS-400, Smiths Medical, Plymouth, MN, USA) at minute 0:00 and every 4 minutes of exercise and at minute 4:00 of recovery.

### Cognitive tests

The following cognitive tests were administered as previously described^13^^,^^14^ and performed in random order: the Digit-Symbol Substitution Test (DSST) that measures primarily processing speed, the Balloon Analogue Risk Task (BART) for risky decision-making prone behaviour and the Psychomotor Vigilance Test (PVT) for attention. The parameters analysed were number of correct and incorrect responses for DSST, total time of test execution, mean earnings and mean pumps for BART, and mean reaction time, performance score and number of lapses for PVT. Before the start of the whole protocol, a baseline session without mask in NX was also performed.

### Visual analogue scales

Before the mask was taken off and, in the case of exercise, with the subject still pedalling, visual analogue scale (VAS) for dyspnoea (100-mm long, where 0 mm was labelled as ‘no difficulty in breathing’ and 100 mm as ‘great difficulty in breathing’) and mask’s discomfort (where 0 mm was labelled as ‘no discomfort’ and 100 mm as ‘very uncomfortable’) were administered. The VAS for dyspnoea was also administered in the no mask condition.

### Statistics

Mixed models were performed to analyse cardiorespiratory parameters (PaCO_2_, PaO_2_, SaO_2_, SpO_2_, pH, [La], [HCO_3_^−^], HR, *f*_R_ and ScO_2_), T_Ty_, parameters of cognitive tests and VAS values. The factors investigated were altitude (NX, HH), mask condition (surgical, FFP2, none), altitude sequence (first NX then HH, first HH then NX) and gender. In addition: (i) for cardiorespiratory parameters and VAS values also exercise (present, absent), rest-exercise sequence (first rest then exercise, first exercise then rest) and interactions of altitude with exercise and of altitude with exercise and mask condition were analysed; (ii) for T_Ty_ additional analysed factors were timepoint during exercise (start, 4th and 8th min after start, stop, 4th min after stop), ear (left, right) and interaction of altitude with timepoint; (iii) for parameters of cognitive tests also rest-exercise sequence, parameter’s value at baseline, progressive number of session (from 1 to 6) and interaction of altitude with mask condition were analysed. Normal distribution of the data was assessed by means of Shapiro–Wilk test and normal Q-Q plots. Generalised linear mixed models (GLMMs) with Gamma distribution were used for ScO_2_ and BART total time of test execution, GLMMs with Poisson distribution for DSST number of correct and incorrect responses and PVT number of lapses, and linear mixed models (LMMs) for all the other parameters. Multiple comparisons were adjusted by means of Holm–Bonferroni correction. SPSS version 27 (IBM Corp., Armonk, NY, USA) was used for statistical analysis and *P* < 0.05 (two-sided) was considered statistically significant. Values of normally distributed parameters are reported as mean ± standard deviation and values of not normally distributed parameters as median (interquartile range), while estimated means of the LMM and GLMM are reported as mean (95% confidence interval, CI).

## Results

### Physiological measurements

**Figure 2:**
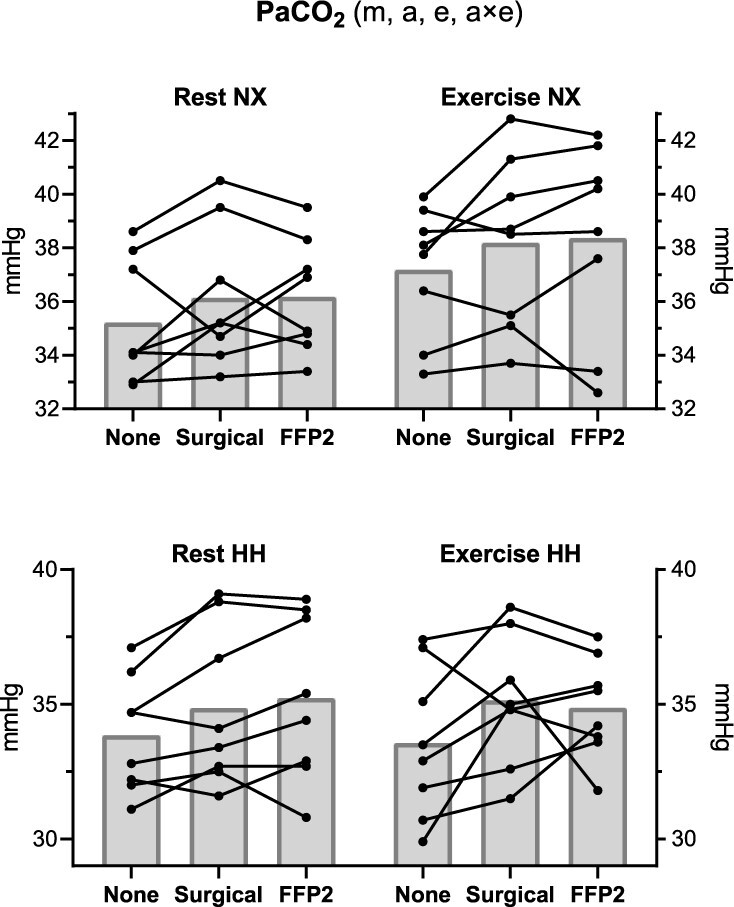
Arterialised carbon dioxide partial pressure (PaCO_2_). Black dots indicate individual values, and grey bars average values. Statistically significant effects of mask (m), altitude (a), exercise (e) and interaction (×) are displayed in the figure heading. NX, normoxia; HH, hypobaric hypoxia.

**Figure 3:**
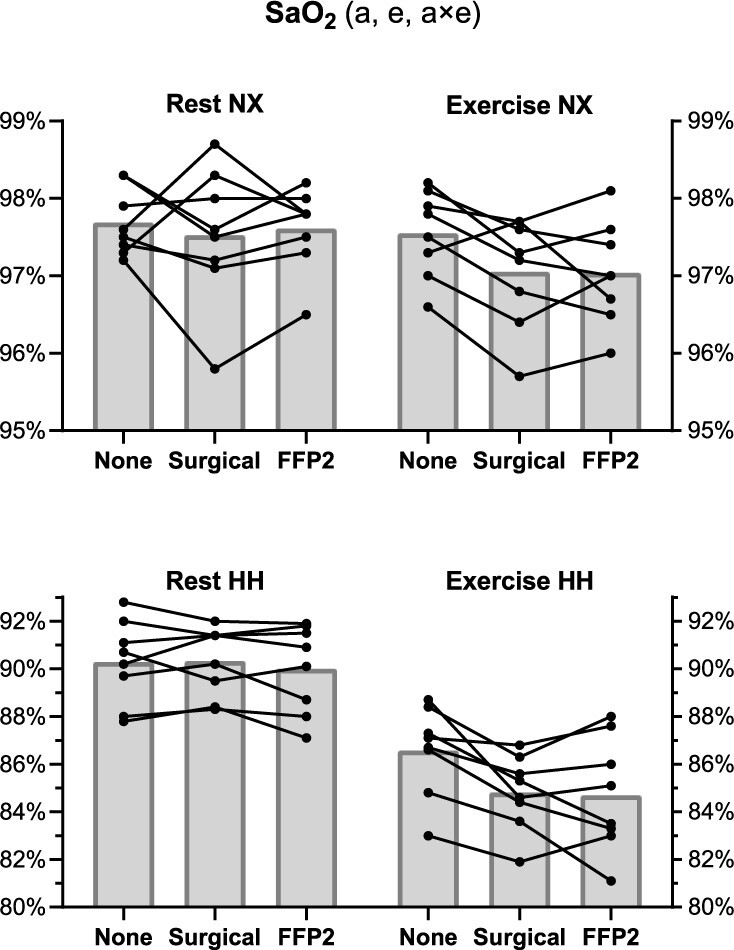
Arterialised oxygen saturation (SaO_2_). Black dots indicate individual values, and grey bars average values. Statistically significant effects of altitude (a), exercise (e) and interaction (×) are displayed in the figure heading. NX, normoxia; HH, hypobaric hypoxia.

Descriptive statistics of the physiological parameters at different altitude, activity (rest and exercise) and mask conditions are shown in Table 1. *P*-values of the factors resulting from the mixed models performed on cardiorespiratory parameters are shown in Table 2. The mask condition had a significant effect only on PaCO_2_ (*P* = 0.016) irrespective of altitude, which was overall 1.1 mmHg (95% CI 0.3–2.0) higher with the surgical and 1.2 mmHg (95% CI 0.3–2.1) higher with FFP2 compared with not wearing a mask. Individual values of PaCO_2_ are shown in Figure 2. During exercise, there was a trend towards lower mean values of SaO_2_ (Figure 3) and PaO_2_ (Supplementary Figure S1) with surgical mask and FFP2 in both NX and HH, although no mask effect was detected (effect of mask condition on SaO_2_ and PaO_2_, *P* = 0.420 and *P* = 1, respectively; effect of the interaction of altitude with exercise and mask condition on SaO_2_ and PaO_2_, *P* = 1 for both). Additional statistically significant effects on cardiorespiratory parameters are reported in the Supplementary Data.

**Table 1:** Descriptive statistics of main parameters

		**Rest**		**Exercise**	
		**NX**	**HH**	**NX**	**HH**
**PaCO**_**2**_ (mmHg) m, a, e, a × e	None	35.2 ± 2.3	33.9 ± 2.1	37.2 ± 2.4	33.6 ± 2.8
	Surgical	36.1 ± 2.6	34.9 ± 2.9	38.2 ± 3.2	35.2 ± 2.4
	FFP2	36.2 ± 2.1	35.2 ± 3.1	38.4 ± 3.6	34.9 ± 1.9
**PaO**_**2**_ (mmHg) a, e, a × e	None	93.7 ± 4.0	58.3 ± 4.7	90.7 ± 5.4	50.2 ± 2.3
	Surgical	93.4 ± 7.1	57.7 ± 3.5	89.4 ± 4.9	48.2 ± 2.1
	FFP2	95.2 ± 4.2	57.4 ± 3.5	89.2 ± 6.1	48.6 ± 2.2
**SaO**_**2**_ (%) a, e, a × e	None	97.7 ± 0.4	90.3 ± 1.8	97.5 ± 0.6	86.6 ± 1.9
	Surgical	97.5 ± 0.9	90.3 ± 1.5	97.1 ± 0.7	84.8 ± 1.6
	FFP2	97.6 ± 0.5	90.0 ± 1.9	97.0 ± 0.7	84.7 ± 2.4
**pH** a	None	7.43 ± 0.03	7.45 ± 0.02	7.42 ± 0.02	7.46 ± 0.02
	Surgical	7.43 ± 0.03	7.45 ± 0.04	7.41 ± 0.03	7.44 ± 0.02
	FFP2	7.43 ± 0.02	7.44 ± 0.02	7.41 ± 0.03	7.45 ± 0.03
**[La]** (mM) e	None	0.8 ± 0.4	0.7 ± 0.3	0.7 ± 0.3	1.0 ± 0.6
	Surgical	0.8 ± 0.4	0.7 ± 0.2	0.9 ± 0.7	1.1 ± 0.9
	FFP2	0.7 ± 0.3	0.7 ± 0.2	0.8 ± 0.3	1.1 ± 0.7
**HR** (min^−1^) a, e	None	67 ± 3	75 ± 7	105 ± 10	120 ± 14
	Surgical	67 ± 4	75 ± 8	105 ± 11	120 ± 16
	FFP2	66 ± 5	72 ± 8	105 ± 7	118 ± 13
***f***_**R**_ (min^−1^) e	None	17 ± 2	16 ± 3	20 ± 4	23 ± 5
	Surgical	18 ± 2	17 ± 2	20 ± 4	22 ± 6
	FFP2	17 ± 3	17 ± 3	20 ± 4	23 ± 6
**SpO**_**2**_ (%) a, e, a × e	None	98.8 ± 0.7	92.4 ± 2.4	98.5 ± 0.5	88.2 ± 2.6
	Surgical	99.0 ± 0.7	92.7 ± 1.3	98.3 ± 0.5	87.4 ± 1.8
	FFP2	98.9 ± 0.7	92.1 ± 1.0	98.3 ± 0.5	87.3 ± 2.1
**ScO**_**2**_ (%) a, a × e	None	65.5 (65.0–69.8)	60.9 (59.6–66.1)	67.2 (66.1–73.0)	60.2 (58.4–63.3)
	Surgical	65.7 (65.0–70.3)	61.4 (59.3–68.1)	68.4 (65.2–72.9)	59.7 (58.3–63.7)
	FFP2	64.9 (64.4–69.4)	60.7 (59.5–64.7)	66.6 (65.4–74.2)	59.4 (57.8–63.3)
**End-exercise T**_**Ty,**_ **left** (°C) a	None			36.3 ± 0.3	36.5 ± 0.4
	Surgical			36.2 ± 0.4	36.4 ± 0.3
	FFP2			36.3 ± 0.3	36.4 ± 0.2
**End-exercise T**_**Ty,**_ **right** (°C) a	None			35.9 ± 0.5	36.4 ± 0.2
	Surgical			35.9 ± 0.4	36.2 ± 0.5
	FFP2			35.9 ± 0.5	36.3 ± 0.2
**Dyspnoea** (0–100)^a^ m, e	None	0 ± 0	0 ± 0	3 ± 6	6 ± 11
	Surgical	13 ± 14	10 ± 9	30 ± 22	34 ± 24
	FFP2	17 ± 17	19 ± 15	44 ± 30	49 ± 31
**Mask’s discomfort** (0–100)^b^ m, e	None	N/A	N/A	N/A	N/A
	Surgical	19 ± 23	18 ± 21	32 ± 24	33 ± 30
	FFP2	33 ± 33	31 ± 32	41 ± 31	42 ± 28

NX, normoxia; HH, hypobaric hypoxia. Effects of mask (m), altitude (a), exercise (e) and interaction (×) are highlighted in the first column. *N* = 8.

^a^0, ‘no difficulty in breathing’; 100 ‘great difficulty in breathing’.

^b^0, ‘no discomfort’; 100, ‘very uncomfortable’.

**Table 2:** *P*-values of the factors resulting from the mixed models performed on each cardiorespiratory parameter

	**Intercept**	**Altitude**	**Exercise**	**Mask**	**Altitude sequence** ^a^	**Rest-exercise sequence** ^b^	**Gender**	**Altitude × exercise**	**Altitude × exercise × mask**
**PaCO**_**2**_	**<0.001**	**<0.001**	**0.039**	**0.016**	0.114	**0.012**	**<0.001**	**0.039**	1.000
**PaO**_**2**_	**<0.001**	**<0.001**	**<0.001**	1.000	1.000	0.639	0.376	**0.032**	1.000
**SaO**_**2**_	**<0.001**	**<0.001**	**<0.001**	0.420	**<0.001**	**<0.001**	**<0.001**	**<0.001**	1.000
**SpO**_**2**_	**<0.001**	**<0.001**	**<0.001**	1.000	1.000	1.000	1.000	**<0.001**	1.000
**pH**	**<0.001**	**<0.001**	0.101	1.000	1.000	1.000	1.000	0.199	1.000
**[La]**	**<0.001**	0.630	**0.043**	1.000	1.000	1.000	1.000	0.339	1.000
**[HCO**_**3**_ ^**−**^**]**	**<0.001**	1.000	1.000	0.695	1.000	1.000	**0.027**	1.000	1.000
**HR**	**<0.001**	**0.003**	**<0.001**	1.000	0.626	1.000	0.229	0.607	1.000
***f***_**R**_	**<0.001**	1.000	**<0.001**	1.000	1.000	1.000	1.000	0.599	1.000
**ScO**_**2**_	**<0.001**	**<0.001**	1.000	1.000	0.788	1.000	1.000	**0.035**	1.000

Linear mixed models were used, except for ScO_2_ a generalised linear mixed model with Gamma distribution was performed. *P*-values are adjusted by means of Holm–Bonferroni correction. *N* = 8.

^a^First 250 m then 3000 m vs first 3000 m then 250 m.

^b^First rest then exercise vs first exercise then rest; ×, interaction.

No effect of mask condition was detected on T_Ty_ (*P* = 0.432). T_Ty_ increased with exercise (*P* < 0.001) from 35.9 °C (95% CI 35.8–36.0) to 36.2 °C (95% CI 36.1–36.3) and did not change 4 min after exercise stopped [36.2 °C (95% CI 36.1–36.2)]. T_Ty_ was higher in HH compared with NX [36.2 °C (95% CI 36.1–36.2) vs 35.9 °C (95% CI 35.8–36.0), *P* < 0.001], in the left ear compared with the right one [36.1 °C (95% CI 36.0–36.1) vs 36.0 °C (95% CI 35.9–36.0), *P* < 0.001] and in females compared with males [36.2 °C (95% CI 36.1–36.3) vs 35.9 °C (95% CI 35.8–36.0), *P* < 0.001].

### Cognitive performance

No effect of altitude and mask conditions was detected for parameters of the cognitive tests (*P* = 1 for all, see Supplementary Tables S1 and S2). BART total time of test execution decreased with each subsequent session (*P* = 0.002), as every session lasted 3% (95% CI 2–5%) less than the previous one. The number of correct responses of DSST was higher for participants who were first in HH and then in NX [59 (95% CI 58–60) vs 57 (95% CI 56–58), *P* = 0.015] in comparison with participants who were in NX and then in HH. For DSST number of correct responses, BART total time of test execution, BART mean earnings and PVT performance score an effect indicating a positive correlation with the value at baseline was detected (*P* = 0.018, *P* < 0.001, *P* = 0.041 and *P* = 0.040, respectively).

### Dyspnoea and discomfort

Perceived dyspnoea and mask’s discomfort were affected by exercise and mask (*P* < 0.001 for both) and were greatest with FFP2, irrespective of altitude. Participants who carried out the exercise before the rest session reported higher dyspnoea [27 (95% CI 17–36) vs 11 (95% CI 1–20), *P* = 0.030] and higher discomfort [50 (95% CI 34–67) vs 12 (95% CI 0–28), *P* = 0.010] compared with participants who carried out the rest before the exercise session.

## Discussion

The main findings of the present study in regard to gas exchange during mask wearing in HH equivalent to 3000-m altitude are that (i) similar to sea level,^6^ there are only small effects on CO_2_ retention (~1 mmHg), and (ii) contrary to our hypothesis, even during exercise the decrease in O_2_ saturation compared with no mask wearing was not clinically relevant, being low at both the group level (−2% SaO_2_ and −1% SpO_2_) and the individual level (range −4 to +1% for both SaO_2_ and SpO_2_). With respect to cognitive function and thermal stress, no influence of mask wearing was detected. This study confirms the results obtained during a simulated commercial flight^9^ and extends them by increasing both altitude and physical activity levels, with applicability ranging from mountain^3^ to aviation^10^ scenarios, not only for passengers but also for crew members performing physical or cognitive tasks.

### Physiological measurements

From a physiological perspective, the observed small modifications in blood gases with face mask use suggest a slight decrease in alveolar ventilation.^19^ Since face masks significantly increase physiological dead space,^20^ there is no need to hypothesize a reduction in total minute ventilation, but only the absence of its compensatory increase. This blunted ventilatory response to an increase in dead space can be explained by the slower inhalation and exhalation patterns reported in young to middle-aged adults,^7^ likely related to the external airway resistance (whose order of magnitude is far from limiting submaximal ventilation^6^) and/or to an avoidance reaction to unpleasant feelings (e.g. bad smell, air jets in the eyes). The assumption of an unchanged total minute ventilation, coupled with the fact that *f*_R_ was not increased, indicates that reduction in tidal volume was negligible, even during exercise. This is in line with most, but not all, studies on submaximal exercise near sea level,^5^ since in some experimental settings tidal volume may have been underestimated by the questionable use of an ergospirometry mask on top of the face mask, as previously criticised.^21^ The stability of the HR is also a strong indirect marker of absence of hypoventilation^22^ or frank apnoeas,^23^ which would have severely perturbed HR response to exercise.

Despite the site for internal temperature monitoring was closer to the face with respect to other studies,^24^^,^^25^ we confirm the absence of thermal stress induced by face masks irrespectively of metabolic rate and extend these results to high altitude. As a secondary result, we confirm the asymmetry in T_Ty_ (left higher than right), previously reported during increases in core temperature.^26^ Due to the proximity and the shared vasculature between the ear and the ipsilateral brain structures,^27^ this asymmetry is in line with previous studies on lateralization of cerebral functions, cerebral blood flow and thus heat exchange.^28^^,^^29^

### Cognitive performance

That cognitive performance was not affected by acute altitude exposure was an expected finding, given that even in short-term HH exposure corresponding to 5000 m above sea level, there was only a slight slowing of the reaction time at the PVT.^13^ Importantly, the combination of mask use and HH seems not to exert any additional detrimental effect on cognitive measurements, reproducing sea-level results.^30–33^ This is in line with the unaffected ScO_2_, which can to some extent explain this finding. Increased cerebral perfusion, ScO_2_ and blood-oxygen-level-dependent signal in response to mask use has been reported,^34^^,^^35^ which fits well with our observed concomitant small increase in PaCO_2._^36^ Collectively, these preliminary findings suggest that cognitive-challenging activities can be safely carried out while wearing a face mask at high altitude.

### Dyspnoea and discomfort

Despite the only minimal change in physiological parameters, perceived dyspnoea increased significantly as previously reported^30^^,^^37^ and can therefore be ascribed to a *nocebo* effect. This is similar to the decrease in perceived physical performance while wearing a mask during cardiopulmonary resuscitation, despite objective performance being unaffected.^38^ Incidentally, perceived dyspnoea and discomfort were higher when exercise was performed first, suggesting a sensitising effect of exercise on mask-related unpleasant feelings, a desensitising effect of the cognitive tests, or both. These subjective factors should not be underestimated because they affect adherence to mask use, which is a key variable of mask effectiveness,^4^ and which can be even more challenging in remote high-altitude environments.

### Limitations

Our results are limited to short-term face mask use, acute exposure to 3000-m altitude, resting to moderate exercise conditions (only rest for cognitive tests, only exercise for T_Ty_) and healthy subjects. Wearing face mask for longer durations during health care work was associated with dyspnoea, fatigue and headache, while hypercapnia and desaturation were still clinically irrelevant.^37^^,^^39^ On the other hand, a longer exposure to high altitude can impair cognitive performance.^14^ Therefore, in future, it would be appropriate to assess the effect of prolonged face mask use in HH with a multi-day study, which after acclimatisation would also allow to safely test at altitudes higher than 3000 m. Despite the apparently low exercise intensity, in HH, 64 ± 7% of the maximal HR predicted for 3000-m altitude^40^ was elicited. While a higher intensity would certainly have increased the respiratory burden imposed by face masks,^6^^,^^33^ their use is not recommended during high-intensity exercise.^1^ Present results may not apply to people with pre-existing health conditions. Of note, patients with chronic obstructive pulmonary disease did not have major changes 6-min walking distance and SpO_2_ with surgical mask at sea level.^41^ In more severe disease states, such as advanced parenchymal or vascular lung disease or decompensated heart failure, regardless of whether masks are worn, exposure to high altitude is *per se* contraindicated.^42^

Although the sample size was enough to detect main predictable cardiorespiratory changes, it was not large enough to provide enough power for more subtle or heterogeneous changes in other parameters, in particular those regarding cognitive function. Even if a larger sample size had revealed a statistical significance of the mask-induced desaturation during exercise in HH, its magnitude would have remained below the commonly accepted clinically significant threshold of −4 percentage points.^43^ With the current sample size, however, cognitive results represent a pilot study and caution should be used when interpreting them.

## Conclusions

Despite surgical masks and FFP2 were associated with higher rates of dyspnoea, their use had no clinically relevant impact on gas exchange at 3000-m altitude both at rest and during moderate exercise, and no detectable effect on cognitive performance at rest. Wearing a surgical mask or an FFP2 can be considered safe for healthy people living, working or spending their leisure time in mountains, high-altitude cities or other hypobaric environments (e.g. aircrafts) up to an altitude of 3000 m.

## Funding

This study was supported by the ‘Fusion Grant’ call, sponsored by the Fondazione Cassa di Risparmio di Bolzano in collaboration with NOI Techpark, Südtiroler Wirtschaftsring and Rete Economia Alto Adige.

## Authors’ contribution

S.R., H.B., H.G. and G.S. conceived and designed the research. G.V., A.M., S.R. and A.R. conducted the experiments. M.F. conceived and provided cognitive tests. T.D.C. and G.V. analysed data. All authors interpreted the results. G.V. wrote the first draft of the manuscript. All authors critically read, amended and approved the manuscript.

## Conflict of interest

The authors have declared no conflicts of interest.

## Data availability

The datasets generated and analysed during the current study are available from the corresponding author on reasonable request.

## CRediT author Statement

Giovanni Vinetti (Data curation-Lead, Formal analysis-Supporting, Investigation-Equal, Methodology-Equal, Project administration-Lead, Visualisation-Lead, Writing—original draft-Lead, Writing—review & editing-Equal), Alessandro Micarelli (Data curation-Supporting, Investigation-Equal, Methodology-Equal, Resources-Equal, Writing—review & editing-Equal), Marika Falla (Data curation-Equal, Methodology-Equal, Resources-Equal, Software-Equal, Writing—review & editing-Equal), Anna Randi (Data curation-Supporting, Investigation-Supporting, Resources-Supporting, Software-Supporting, Writing—review & editing-Supporting), Tomas Dal Cappello (Data curation-Supporting, Formal analysis-Lead, Writing—review & editing-Equal), Hannes Gatterer (Conceptualization-Equal, Funding acquisition-Supporting, Methodology-Equal, Supervision-Equal, Writing—review & editing-Equal), Hermann Brugger (Conceptualization-Equal, Funding acquisition-Supporting, Methodology-Equal, Supervision-Equal, Writing—review & editing-Equal), Giacomo Strapazzon (Conceptualization-Equal, Funding acquisition-Supporting, Supervision-Equal, Writing—review & editing-Equal) and Simon Rauch (Conceptualization-Lead, Funding acquisition-Lead, Investigation-Equal, Project administration-Supporting, Supervision-Equal, Writing—review & editing-Equal)

## Supplementary Material

Supplementary_Data_JTM_R1_taad031
