# Supplementary material for: Surgical masks and filtering facepiece class 2 respirators (FFP2) have no major physiological effects at rest and during moderate exercise at 3000-m altitude: a randomised controlled trial
Source: J Travel Med. 2023 Mar 6;30(5):taad031. doi: 10.1093/jtm/taad031 (PMC10481409; doi:10.1093/jtm/taad031)
Supplement: Supplementary_Data_JTM_R1_taad031 [file supplementary_data_jtm_r1_taad031.docx]

Supplementary data

# Additional statistically significant effects on cardiorespiratory parameters

## Gender

PaCO_2_ and [HCO_3_^−^] were higher in males compared to females [37.8 (95% CI 37.2-38.5) vs 33.6 (95% CI 33.0-34.3), p<0.001, mmHg and 25 (95% CI 24-26) vs 23 (95% CI 22-24) mM, p=0.027, respectively]. SaO_2_ was higher in females compared to males [93.0 (95% CI 92.7-93.2) vs 92.2 (95% CI 91.9-92.5) %, p<0.001].

## Altitude sequence

Participants who were first in NX and then in HH had higher SaO_2_ [93.0 (95% CI 92.7-93.2) vs 92.2 (95% CI 91.9-92.4) %, p<0.001] compared to participants who were first in HH and then in NX.

## Rest-exercise sequence

Participants who carried out the exercise before the rest session had a higher PaCO_2_ [36.5 (95% CI 35.9-37.1) vs 35.0 (95% CI 34.4-35.6) mmHg, p=0.012] and a lower SaO_2_ [92.2 (95% CI 92.0-92.5) vs 92.9 (95% CI 92.7-93.2) %, p<0.001] compared to participants who carried out the rest session before the exercise.

## Interaction of altitude with exercise

PaCO_2_ and ScO_2_ were higher in exercise compared to rest in NX, but not in HH [for PaCO_2_, in NX 35.8 (95% CI 35.1-36.6) mmHg at rest vs 37.9 (95% CI 37.1-38.7) mmHg in exercise and in HH 34.6 (95% CI 33.9-35.4) mmHg at rest vs 34.5 (95% CI 33.7-35.3) mmHg in exercise; for ScO_2_, in NX 67.8 (95% CI 62.3-73.7) % at rest vs 69.6 (95% CI 64.0-75.6) % in exercise and in HH 64.0 (95% CI 58.8-69.6) % at rest vs 63.0 (95% CI 58.0-68.6) % in exercise].

PaO_2_ and SaO_2_ had a more pronounced decrease from rest to exercise in HH than in NX [for PaO_2_, in NX 94.1 (95% CI 92.3-96.0) mmHg at rest vs 89.8 (95% CI 87.9-91.6) mmHg in exercise and in HH 57.8 (95% CI 56.0-59.7) mmHg at rest vs 49.0 (95% CI 47.1-50.9) mmHg in exercise; for SaO_2_, in NX 97.6 (95% CI 97.4-97.8) % at rest vs 97.2 (95% CI 97.1-97.4) % in exercise and in HH 90.2 (95% CI 89.6-90.8) % at rest vs 85.4 (95% CI 84.6-86.1) % in exercise].

SpO_2_ decreased from rest to exercise in HH but not in NX [in NX 98.9 (95% CI 97.9-99.8) % at rest vs 98.4 (95% CI 97.4-99.3) % in exercise and in HH 92.4 (95% CI 91.4-93.3) % at rest vs 87.6 (95% CI 86.7-88.6) % in exercise].

**Table S1.** Descriptive statistics of the parameters of the cognitive tests at baseline and in normoxia and hypobaric hypoxia with different mask conditions. Values are reported as mean ± standard deviation if the parameter is normally distributed, otherwise as median (interquartile range). N=8.

| **Test** | **Parameter** | **Baseline** | **Normoxia** | | | **Hypobaric hypoxia** | | |
| --- | --- | --- | --- | --- | --- | --- | --- | --- |
|  |  |  | **No mask** | **Surgical** | **FFP2** | **No mask** | **Surgical** | **FFP2** |
|  |  |  |  |  |  |  |  |  |
| DSST | Number of correct responses | 54 (51 - 55) | 57 (55 - 60) | 59 (58 - 61) | 58 (55 - 61) | 59 (56 - 59) | 57 (56 - 60) | 57 (56 - 61) |
|  | Number of incorrect responses | 1 (0 - 2) | 1 (0 - 3) | 1 (1 - 1) | 1 (0 - 2) | 2 (0 - 2) | 1 (0 - 1) | 1 (0 - 3) |
| BART | Time of test execution (s) | 186 (141 - 225) | 175 (143 - 206) | 156 (137 - 190) | 171 (141 - 200) | 160 (138 - 210) | 171 (140 - 223) | 180 (141 - 201) |
|  | Mean earnings | 8.8 ± 1.7 | 9.2 ± 1.8 | 8.9 ± 1.8 | 9.2 ± 1.8 | 9.1 ± 1.8 | 9.1 ± 1.8 | 9.0 ± 1.7 |
|  | Mean pumps | 7.8 ± 1.7 | 8.2 ± 1.8 | 8.0 ± 1.8 | 8.2 ± 1.8 | 8.1 ± 1.8 | 8.1 ± 1.8 | 8.0 ± 1.7 |
| PVT | Mean reaction time (ms) | 263 ± 17 | 258 ± 13 | 258 ± 16 | 258 ± 15 | 257 ± 12 | 262 ± 14 | 262 ± 13 |
|  | Performance score | 0.94 ± 0.06 | 0.91 ± 0.09 | 0.92 ± 0.05 | 0.90 ± 0.06 | 0.93 ± 0.06 | 0.90 ± 0.09 | 0.91 ± 0.05 |
|  | Number of lapses | 1 (0 - 1) | 1 (0 - 3) | 2 (1 - 2) | 1 (1 - 2) | 0 (0 - 1) | 1 (1 - 2) | 2 (0 - 2) |
|  |  |  |  |  |  |  |  |  |

BART, Balloon Analogue Risk Task; DSST, Digit-Symbol Substitution Test; FFP2, filtering facepiece class 2 respirator; ms, milliseconds; PVT, Psychomotor Vigilance Test; s, seconds.

**Table S2.** P-values of the factors resulting from the mixed models performed on each parameter of the cognitive tests. Linear mixed models were used, except for the number of correct and incorrect responses of the DSST and the number of lapses of the PVT, for which generalised linear mixed models (GLMMs) with Poisson distribution were performed, and except for the time of test execution of the BART, for which a GLMM with Gamma distribution was used. P-values are adjusted by means of Holm-Bonferroni correction. N=8 except *; × denotes interaction.

| **Test** | **Parameter** | **Intercept** | **Altitude** | **Mask** | **Altitude sequence** | **Rest-exercise sequence** | **Gender** | **Parameter's value at baseline** | **Session** | **Altitude × mask** |
| --- | --- | --- | --- | --- | --- | --- | --- | --- | --- | --- |
|  |  |  |  |  |  |  |  |  |  |  |
|  |  |  |  |  |  |  |  |  |  |  |
| DSST | Number of correct responses | **0.004** | 1.000 | 1.000 | **0.015** | 0.149 | 1.000 | **0.018** | 1.000 | 1.000 |
|  | Number of incorrect responses | 0.709 | 1.000 | 1.000 | 0.089 | 1.000 | 1.000 | 0.312 | 0.188 | 1.000 |
| BART | Time of test execution | **<0.001** | 1.000 | 1.000 | 1.000 | 1.000 | 1.000 | **<0.001** | **0.002** | 0.706 |
|  | Mean earnings * | 1.000 | 1.000 | 1.000 | 1.000 | 1.000 | 0.522 | **0.041** | 1.000 | 1.000 |
|  | Mean pumps * | 1.000 | 1.000 | 1.000 | 1.000 | 1.000 | 0.712 | 0.097 | 1.000 | 1.000 |
| PVT | Mean reaction time | 0.408 | 1.000 | 1.000 | 1.000 | 1.000 | 1.000 | 0.277 | 1.000 | 1.000 |
|  | Performance score ^#^ | 1.000 | 1.000 | 1.000 | 1.000 | 1.000 | 1.000 | **0.040** | 1.000 | 1.000 |
|  | Number of lapses | 1.000 | 1.000 | 1.000 | 0.365 | 1.000 | 1.000 | 1.000 | 1.000 | 1.000 |
|  |  |  |  |  |  |  |  |  |  |  |

* one participant excluded from the indicated analysis because outlier; ^#^ arcsin transformation was used to obtain Normal distribution.

BART, Balloon Analogue Risk Task; DSST, Digit-Symbol Substitution Test; PVT, Psychomotor Vigilance Test.

**Figure S1.** Arterialised oxygen partial pressure (PaO_2_). Black dots indicate individual values, and grey bars average values. Statistically significant effects of altitude (a), exercise (e), and interaction (×) are displayed in the figure heading. NX, normoxia; HH, hypobaric hypoxia.
